# C-reactive protein is associated with postoperative outcomes in patients with intestinal Behçet’s disease

**DOI:** 10.1186/s12876-021-01922-2

**Published:** 2021-10-07

**Authors:** Eun Ae Kang, Jung Won Park, Yehyun Park, Soo Jung Park, Tae Il Kim, Won Ho Kim, Min Soo Cho, Jae Hee Cheon

**Affiliations:** 1 Gastroenterology and Department of Internal Medicine, Yonsei University College of Medicine, 50-1 Yonsei-ro, Seodaemun-gu, Seoul, 03722 Republic of Korea; 2 Division of Colon and Rectal Surgery, Department of Surgery, Yonsei University College of Medicine, 50-1 Yonsei-ro, Seodaemun-gu, Seoul, 03722 Republic of Korea

**Keywords:** Intestinal Behçet’s disease, surgery, CRP, prognosis, complication

## Abstract

**Background:**

Patients with intestinal Behçet’s disease (BD) frequently undergo intestinal resections, which significantly affects postoperative morbidity and mortality. The aim of this study was to identify the association between C-reactive protein (CRP) levels and postoperative outcomes in patients with intestinal BD who underwent surgical bowel resection.

**Methods:**

Patients who were diagnosed with intestinal BD and underwent intestinal surgery due to BD at Severance Hospital between November 2005 and April 2018 were retrospectively investigated. Clinical relapse was defined as a disease activity index of BD (DAIBD) > 40, existence of newly added medications, re-hospitalization, or re-operation related to intestinal BD. The relationship between CRP level and postoperative outcomes was analyzed, and a receiver operating characteristic (ROC) curve was drawn to specify a cut-off value.

**Results:**

Ninety patients with intestinal BD were included. Among them, 44 were male (48.9%), and the median age at diagnosis was 38 years (range, 11–69 years). The median total disease follow-up duration was 130 months (range, 3–460 months). Forty patients (44.4%) underwent laparoscopic surgery. A higher CRP level immediately after surgery was significantly associated with postoperative complications (OR 1.01, 95% CI 1.004–1.018, *p* < 0.01), re-operation (hazard ratio [HR] 1.01, 95% CI 1.005–1.020, *p* < 0.01), and re-admission (HR 1.01, 95% CI 1.006–1.017 *p *< 0.01). The ROC curve showed that CRP predicts the risk of postoperative complications (*p* < 0.01) at a cut-off value of 41.9% with a sensitivity of 60.0% and specificity of 67.7%.

**Conclusions:**

Postoperative CRP levels in patients with intestinal BD undergoing surgical resection were associated with postoperative outcomes.

**Supplementary Information:**

The online version contains supplementary material available at 10.1186/s12876-021-01922-2.

## Background

Behçet’s disease (BD) is caused by chronic vascular inflammation in multiple organs and is characterized by oral and genital ulcers, uveitis, arthritis, skin rash, vasculitis, neurologic manifestations, and/or gastrointestinal inflammation [1, 2]. The etiology of BD remains unclear, but several genetic factors and human leukocyte antigen (HLA)-B*51 are well known to be associated with BD development [3–5]. Environmental factors and immunological dysregulation also play a role in pathogenesis. BD is more prevalent in the Middle East and East Asia, including South Korea [6, 7]. There is no single diagnostic test for BD; however, the following diagnostic criteria for BD are commonly used: presence of oral ulcers and any two out of genital ulcers, eye lesions, skin lesions, and positive pathergy test [8].

BD involvement in the gastrointestinal tract, so called “intestinal BD,” is characterized by typical deep ulcerative lesions in the ileocecal valve area based on colonoscopy. Patients with intestinal BD may have significant gastrointestinal symptoms, such as abdominal pain, diarrhea, and gastrointestinal bleeding, but some patients are asymptomatic with a quiescent disease status. Intestinal BD is rare but is more prevalent in East Asia, including Korea and Japan, than in other regions [9]. Intestinal BD may cause severe complications, such as perforation or massive gastrointestinal bleeding, requiring intestinal resection. Intestinal BD is significantly associated with morbidity and mortality in patients with BD.

Intestinal BD can reoccur after intestinal surgery with a chronic, relapsing disease course and may require anti-tumor necrosis factor (TNF) therapy or re-operation. Postoperative outcomes, including complications and relapse, are critical in the prognosis of BD [10, 11]. Early detection of complications and their appropriate management can improve patient prognosis. Therefore, it is important to promptly assess the possibility of poor outcomes after surgery for intestinal BD. C-reactive protein (CRP) is a representative serological marker of inflammation. However, there is still little research on whether postoperative CRP levels and the degree of CRP reduction are associated with postoperative prognosis, particularly in intestinal BD [12, 13]. Herein, we aimed to identify the association between CRP levels and postoperative outcomes in patients with BD undergoing surgical bowel resection.

## Methods

### Study population

This study was a retrospective observational study that enrolled patients with intestinal BD who underwent intestinal surgery due to complications or refractoriness of intestinal BD in a single tertiary hospital between November 2005 and April 2018. Electronic medical records were retrospectively reviewed. Patients with intestinal BD who underwent surgery unrelated to intestinal BD were excluded. Clinical factors, including age at diagnosis, sex, prior abdominal surgical history, and history of perioperative medical treatment, were investigated. Disease duration, intestinal symptoms, and disease activity index of BD (DAIBD) prior to surgery were also evaluated. Laboratory findings, such as CRP, albumin, and hemoglobin levels, within 5 days after surgery were also investigated.

### Definitions and subtypes of intestinal BD

Intestinal BD was diagnosed if patients with systemic BD had ulcerations in the gastrointestinal tract, especially in the ileocecal area. Clinical symptoms, radiologic, endoscopic, and histologic findings based on the diagnostic criteria for BD were used for diagnosis [14–16]. If patients had typical intestinal ulcers in the ileocecal area and was also diagnosed as having systemic BD, these cases were regarded as “definite” intestinal BD. If patients had typical intestinal ulcers but only had oral ulcers or had atypical intestinal ulcers but was diagnosed as having systemic BD, it was defined as “probable” intestinal BD. If patients had only typical intestinal ulcers without systemic manifestations of BD or had atypical intestinal ulcers and oral ulcers only, we defined it as “suspected” intestinal BD [16]. Typical lesions were defined as deep, oval-shaped, and well-demarcated ulcers less than five in the ileocecal region [16, 17].

### Outcomes

Postoperative outcomes, including the presence of postoperative complications within one month and clinical relapse after intestinal surgery, were investigated. Postoperative complications included anastomotic leak, intra-abdominal abscess, fistula, wound infection, bleeding, perforation, other infections, and wound dehiscence. Clinical relapse was defined as an increase in DAIBD to > 40, newly added medications due to aggravation of intestinal BD, re-admission due to symptomatic aggravations, or cases of re-operation related to intestinal BD [18]. DAIBD consists of a general sense of well-being for a week (0 to 40, add 10 points as severity increases), presence of fever (if yes, add 10 points), abdominal mass (if yes, add 10 points), abdominal tenderness (if yes, add 10 or 20 points according to severity), extra-intestinal manifestations (add 5 points per item; oral ulcer, genital ulcer, eye lesion, skin lesion, arthralgia; add 15 points for vascular or central nervous system involvement), abdominal pain for one week (0 to 80, add 20 points according to severity), intestinal complications (add 10 points per item; fistula, perforation, abscess, or intestinal obstruction), and liquid stool in a week (add 10 points) [19].

Serum CRP levels (mg/L) were measured within five days after surgery. Follow-up CRP levels before discharge were measured repeatedly. We investigated the hazard ratio (HR) of CRP levels for postoperative outcomes in patients with intestinal BD.

### Ethical considerations

The need for informed consent was waived due to the retrospective nature of the observational study. The study protocol was approved by the institutional review board of Severance Hospital. Personal information data were anonymized. All authors had access to the data of this study and approved the final version of the manuscript (institutional review board number: 4-2021-0078).

### Statistical analysis

Categorical variables were analyzed using the χ^2^ test, and continuous variables were analyzed using Student’s t-test. The relationship between CRP level and postoperative outcomes of intestinal BD was measured using logistic regression analysis. Other clinical factors were identified by multivariate analysis using Cox proportional hazard regression model. Furthermore, an area under the receiver operating characteristic curve was drawn to evaluate the accuracy of the results and to specify a cut-off value. Statistical significance was set at *p* < 0.05. All statistical analyses were performed using SPSS software version 25.0 for Windows (SPSS Inc., Chicago, IL, USA).

## Results

### Baseline characteristics of study population

**Fig. 1:**
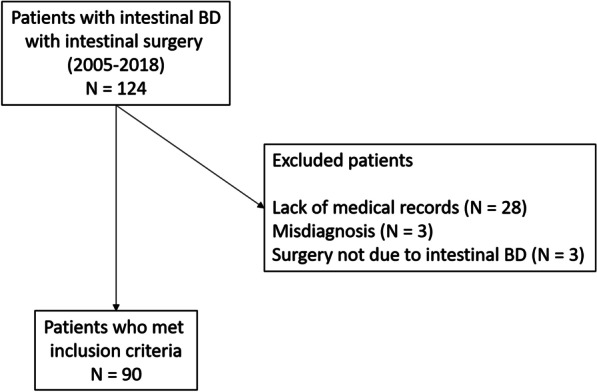
Patients who met inclusion criteria

A total of 124 patients with intestinal BD were screened. Patients whose diagnosis was incorrectly recorded in medical records were excluded (n = 28), and patients whose diagnosis was changed to another inflammatory disease during follow-up were excluded (n = 3). Patients who underwent surgery for reasons other than BD were also excluded from the final analysis (n = 3). Finally, 90 patients with BD who underwent intestinal surgery were investigated (Fig. 1). Forty-four (48.9%) had a median age of 38 years (range, 11–69 years). Median disease follow-up duration was 11.8 years (0.3–38.4 years). Detailed information on the use of perioperative medications, including 5-aminosalicylic acids (5-ASA), immunomodulators (azathioprine, 6-mercaptopurine, methotrexate), colchicine, corticosteroids, and anti-TNF agents, are shown in Table 1. A laparoscopic approach was performed in 40 patients (44.4%). Emergent surgery was performed in 13 cases, mainly due to peritonitis and perforation. The CRP level was measured at a median of 2 (1–4) days after surgery. The number of patients and CRP levels on each day for which CRP was measured after surgery were shown in Additional file 1:  Table S1.

**Table 1:** Baseline characteristics of study population

Patient characteristics	Patients (n = 90)
Male (number, %)	44 (48.9%)
Age at diagnosis (median, range; years)	38 (11–69)
Disease follow-up period (median, range; years)	11.8 (0.3–38.4)
Duration between diagnosis and surgery (median, range; months)	23 (0–217)
Subtype of disease	
Definite	68 (75.6%)
Probable	2 (2.2%)
Suspected	20 (22.2%)
Oral ulcers	66 (73.3%)
Genital ulcers	31 (34.4%)
Skin manifestations	30 (33.3%)
Typical intestinal ulcers	86 (95.6%)
Fistula / abscess	11 (12.2%)/4 (4.4%)
Perioperative medical treatment (number; %)	
5-aminosalicylic acids	86 (95.6%)
Immunomodulator	53 (58.9%)
Azathioprine	43 (47.8%)
6-Mercaptopurine	3 (3.3%)
Methotrexate	7 (7.8%)
Colchicine	45 (50.0%)
Corticosteroids	74 (82.2%)
Anti-TNF antagonist	12 (13.3%)
Surgical technique (number; %)	
Laparoscopy	40 (44.4%)
Open	50 (55.6%)
Emergent surgery	13 (14.4%)
CRP (mg/L)	57.0 ± 71.2
Day of CRP measurement after surgery (median, interquartile range; days)	2 (1–4)

*CRP* C-reactive protein, *n* number, *TNF* tumor necrosis factor

### Postoperative complications and clinical relapse after intestinal surgery in patients with intestinal BD

Postoperative complications within one month after surgery occurred in 25 patients (27.8%), and events occurred in a total of 32 patients (35.6%). Postoperative complications included anastomotic leak, abscess formation, fistula, wound infection, ileus, bleeding, perforation, and other infections. Clinical relapse occurred in 52 patients (57.8%), and the total number of events was 78 (Table 2). Surgical method, such as open surgery or laparoscopic surgery, did not affect the clinical relapse (*p* = 0.42), re-operation (*p* = 0.23), re-admission (*p* = 0.96), or postoperative complications (*p* = 0.14) rates. Furthermore, whether surgery was emergent or elective also did not affect outcomes (clinical relapse, *p* = 0.13; postoperative complications, *p* = 0.11).

**Table 2:** Postoperative complications and clinical relapse in patients with intestinal Behçet’s disease after undergoing intestinal surgery

	Number (%)
*Postoperative complications*	
Total (events/patients, number)	32/25 (27.8%)
Anastomosis leak	8 (25.0%)
Intra-abdominal abscess	6 (18.8%)
Fistula	5 (15.6%)
Wound infection	5 (15.6%)
Ileus	2 (6.2%)
Bleeding	3 (9.4%)
Perforation	2 (6.2%)
Other infection	1 (3.1%)
Wound dehiscence	0 (0.0%)
*Clinical relapse*	
Total (events/patients, number)	76/52 (57.8%)
DAIBD > 40 or additional medications needed^*^	5 (6.6%)
Re-operation	24 (31.6%)
Re-admission	47 (61.8%)

*Cases in which disease activity is increased, only new drugs are added without surgery or hospitalization

*DAIBD* disease activity index of intestinal Behçet’s disease

## Risk factors for postoperative outcomes, including postoperative CRP levels

Mean CRP levels between patients with or without postoperative complications differed significantly (99.44 mg/L vs. 41.95 mg/L, *p* = 0.01). Patients who were readmitted after surgery had higher CRP levels than those who were not (73.40 mg/L vs. 41.72 mg/L, *p* = 0.03).

Risk factors for postoperative outcomes in patients with BD were analyzed using Cox proportional hazard regression analysis. The median time to relapse was 49 months (interquartile range, 13.3–115.3). A higher postoperative CRP level was an independent risk factor for postoperative complications and clinical relapse, including re-operation and re-admission (Table 3). The association between CRP levels and postoperative outcomes was statistically significant when adjusted for age, sex, and perioperative use of medications. A subgroup analysis that included only patients with CRP levels within 4 days after surgery showed similar results in terms of their association with postoperative outcomes; postoperative complications (HR 1.01 (1.002–1.016), *p* = 0.02), readmission (HR 1.01 (1.001–1.013), *p* = 0.02), and reoperation (HR 1.01 (1.001–1.017), *p* = 0.03). Anti-TNF agents tended to lower postoperative complications and relapses but were not statistically significant. Corticosteroid use was associated with postoperative complications (HR 4.36 (1.21–15.68), *p* = 0.02) and re-operation (HR 3.31 (1.15–9.56), *p* = 0.03). Typical volcanic intestinal ulcers and intestinal fistulas were related to clinical relapse, especially re-admission (Additional file 1: Table S2).

**Table 3:** Association of postoperative C-reactive protein levels with postoperative complications and clinical relapse in patients with intestinal Behçet’s disease

	Crude HR (95% CI)	*p*-value	*Adjusted HR (95% CI)	*p*-value
Postoperative complication	1.01 (1.005–1.016)	< 0.01	1.01 (1.004–1.018)	0.01
Clinical relapse	1.01 (1.006–1.013)	< 0.01	1.01 (1.005–1.016)	< 0.01
Re-operation	1.01 (1.006–1.018)	< 0.01	1.01 (1.005–1.020)	< 0.01
Re-admission	1.01 (1.006–1.015)	< 0.01	1.01 (1.006–1.017)	< 0.01

*Adjustment for age at diagnosis, sex, perioperative medication use including anti-TNF agents, immunomodulators, and corticosteroids

*CI* confidence interval, *HR* hazard ratio

### Predictive power and cut-off value of CRP for postoperative outcomes in intestinal BD

**Fig. 2:**
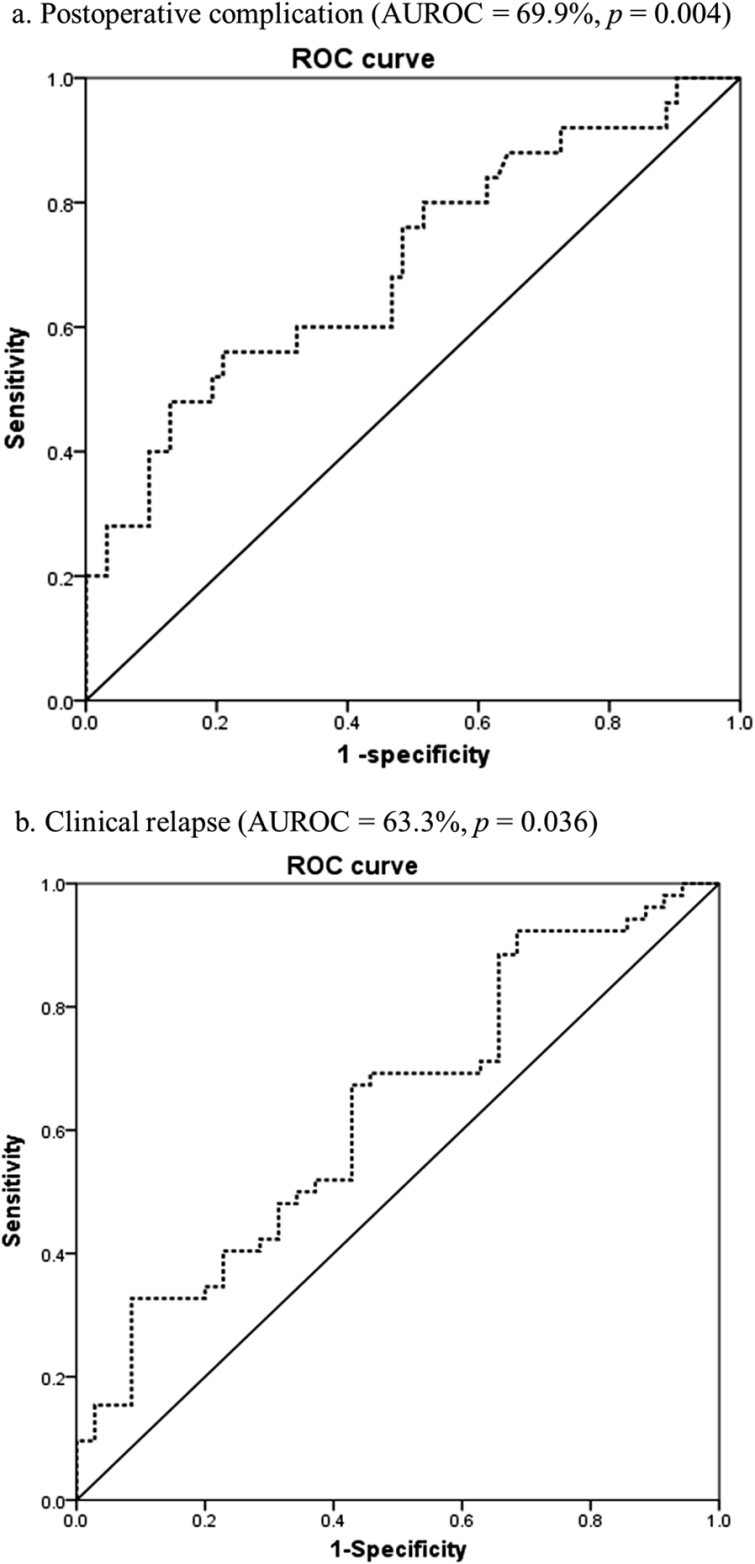
Area under the curve (AUC) of predictive value of CRP level for postoperative complications (**a**) and clinical relapse (**b**) in patients with intestinal BD

We evaluated the predictive power of CRP levels for postoperative outcomes in patients with intestinal BD. As shown in Table 4; Fig. 2, the area under the curve (AUC) of postoperative complications was 69.9%, and the sensitivity was 60.0%. The AUC of clinical relapse, including re-operation and re-admission, was 63.3%, and the sensitivity was 51.9%.

**Table 4:** Area under the curve for postoperative complications and clinical relapse

	AUC (%)	*p*-value	Threshold (mg/L)	Sensitivity (%)	Specificity (%)
Postoperative complications	69.9	0.004	41.9	60.0	67.7
Clinical relapse	63.3	0.036	28.6	51.9	60.0
Re-operation	60.6	0.067	28.2	44.4	50.0
Re-admission	61.4	0.067	41.9	50.0	70.7

*AUC* area under the curve

## Discussion

 BD is a systemic vasculitis that involves several organs, including the mouth, eyes, skin, nervous system, and gastrointestinal tract. Intestinal BD is an important subtype of BD that can cause serious morbidity and poor quality of life in patients with BD. Intestinal BD is characterized by chronic and recurrent inflammation and deep ulcerative lesions of the gastrointestinal tract, especially in the terminal ileum and ileocecal valve [20, 21]. Suspected intestinal BD was defined as having a typical intestinal ulcer with no systemic symptoms or an atypical intestinal ulcer with only oral ulcers. Many gastroenterologists consider intestinal BD when intestinal ulcers are typical, even if systemic symptoms are not completely satisfactory for systemic BD [22–24]. Since we have focused on the postoperative outcome of intestinal BD rather than systemic BD, the probable and suspected cases of intestinal BD should also be included in the analysis. Intestinal BD, similar to other inflammatory bowel diseases, such as CD and ulcerative colitis, is usually treated with 5-ASA, corticosteroids, immunomodulators, and/or anti-TNF agents [25–27]. However, intestinal BD may reoccur or worsen due to refractoriness or loss of response to medications and may require surgical intervention [2]. Few studies have examined the surgical outcomes of intestinal BD; however, those studies that have been published showed that a large number of patients can experience recurrence after surgery and ultimately undergo stoma [11]. Patients with intestinal BD may experience postoperative complications and recurrence more frequently than patients with other intestinal diseases, which necessitates the development of an appropriate predictor of postoperative prognosis. Early recurrence can occur within one month after surgery. Therefore, high-risk groups must be promptly identified and appropriately treated.

The present study aimed to investigate the role of CRP as a predictive marker of postoperative complications and clinical relapse in intestinal BD. CRP levels can be elevated in patients with acute inflammation or infection. The relationship between postoperative CRP levels immediately (within 5 days) after surgery and postoperative outcomes was analyzed. According to public insurance standards, CRP levels cannot be frequently measured in Korea within 3 to 4 days. Therefore, the criteria for postoperative CRP levels were defined within 5 days due to clinical limitations in verifying CRP levels. The CRP level was measured at a median of one postoperative days, which did not differ significantly between patients. Our data demonstrated that the elevation of postoperative CRP levels was associated with a poorer prognosis for postoperative complications and recurrence. The association between CRP levels and poor postoperative outcomes was also statistically significant when we included only patients with CRP levels within 4 days after surgery. However, since CRP levels were low on day 5 after surgery, it is recommended to measure CRP levels early to predict the prognosis after surgery. Emergency surgery and the use of drugs, such as anti-TNF agents, did not affect these results and confirmed that CRP is an independent prognostic factor for postoperative outcomes in intestinal BD. According to Additional file 1: Tables S2, corticosteroid use was associated with postoperative complications and re-operation. Typical volcanic intestinal ulcers and intestinal fistulas were related to clinical relapse, especially re-admission. Typical bowel lesions or fistulas were inversely correlated with clinical recurrence. This indicated that typical lesions can certainly be treated with surgery.

According to a Japanese study on BD, a large number of BD patients experience surgery and recurrence within 2 years, and the cumulative recurrence rate reaches 30–75%. In Korea, the cumulative recurrence rate within 2 years and 5 years after surgery is 12.5% and 22.2–31%, respectively [12]. These findings support our findings. In addition, long-term comparisons of patients with intestinal BD who had surgery or not have shown that patients with ileal and ocular lesions had a higher surgical emergency and experienced frequent relapses after surgery [10].

Long-term clinical outcomes and prognostic factors after intestinal surgery have been studied [28, 29]. The presence of deep ulcers, fistula, abscess, leakage of the anastomosis site, and postoperative use of corticosteroids have been suggested to be risk factors [10, 30, 31]. CRP levels over 4.4 mg/dL were a risk factor for relapse [12]. Mucosal healing can reduce the risk of surgery and recurrence of intestinal BD [32]. A previous study showed that early preemptive surgery had a better prognosis than surgery due to disease aggravation or complications [33]. The postoperative use of azathioprine can reduce clinical relapse [34]. Anti-TNF agents can also improve the clinical course of intestinal BD compared with the use of corticosteroids [25, 35]. Furthermore, emergent surgery and elevation of postoperative erythrocyte sedimentation rate were prognostic factors for re-operation within 6 months in intestinal BD [36]. However, there are no studies on the predictors of early recurrence or complications after surgery for intestinal BD. To date, there is little evidence of clear factors or biomarkers for predicting postoperative prognosis in intestinal BD. To the best of our knowledge, this is the first study to investigate the role of CRP in postoperative complications and recurrence in intestinal BD.

 Several studies have analyzed predictors of recurrence and postoperative complications in CD. A prospective cohort study reported that male sex, cigarette smoking, and history of intestinal resection were risk factors for recurrence of CD [37]. Disease behavior, such as penetrating or structuring type, is a well-established risk factor for postoperative recurrence [38–40]. Perianal disease is also accepted as a risk factor for recurrence and poor prognosis [41]. Interestingly, a higher CRP level was shown to be a risk factor for poor prognosis of CD [42–44], and CRP level was reported to be associated with early postoperative surgical outcomes, which is consistent with our findings [45]. However, surgical predictors such as the type of anastomosis, surgical methods, and histological findings of surgical margins were not associated with postoperative outcomes. Intestinal resection for CD or intestinal BD is not curative because of the chronic and relapsing disease course of the disease. Therefore, postoperative complications and recurrence should be carefully monitored to improve the prognosis.

Emergent surgery is expected to have a higher risk, but there was no significant effect due to the small number of patients in this study. However, elective preemptive surgery may be more helpful in reducing postoperative complications than emergency surgery, and patients with elevated CRP levels after surgery should be carefully monitored for the occurrence of complications and relapse. Since CRP is an easily measurable marker, it has high clinical utility. However, several clinical situations need to be considered. In particular, since the predictability of long-term clinical recurrence is low, an individualized follow-up plan should be determined considering several known prognostic factors.

This study has several limitations. The predictive power and sensitivity of postoperative CRP levels were not high, probably because of the small sample size. CRP may be associated with other systemic infections or residual inflammation after surgery. Therefore, there is a continuing need for research on specific markers to predict the postoperative outcome of intestinal BD. Selection bias may exist because of the retrospective nature of the study. However, CRP may be a candidate predictive marker when referring to several previous studies and considering the high likelihood of recurrence and re-operation after the first surgery for intestinal BD. The usefulness of CRP as a surrogate marker has been reported in previous studies [46]. Our results appear to be meaningful, since we adjusted for emergency surgery and perioperative medications. Postoperative CRP levels were defined within 5 days as there were no previous studies that discussed the best time to measure postoperative CRP to predict postoperative outcomes. CRP levels on day 5 were relatively low compared with those on days 1–4. However, similar results and statistical significance were obtained when patients were included within 5 and 4 days. Therefore, we included all patients with CRP levels within 5 days, and future studies are needed to find a meaningfully optimal time for postoperative CRP measurement. Since this was a retrospective study, we have limited data on fecal calprotectin. Fecal calprotectin can be a surrogate marker of remission in patients with intestinal BD [47]. Future prospective studies are needed to confirm the usefulness of CRP in predicting postoperative outcomes.

## Conclusions

Despite these limitations, this is the first study to focus on postoperative CRP levels, short-term postoperative complications, and clinical relapse. A few studies have reported the role of CRP at diagnosis in determining long-term clinical outcomes and prognosis. Postoperative CRP levels in intestinal BD patients undergoing intestinal surgery are also correlated with early postoperative outcomes. Regardless of whether it was an emergency surgery or what medications were used before surgery, physicians should measure CRP levels after surgery, select the appropriate medications, and perform a careful follow-up taking into account the risk of complications and recurrence. CRP is relatively inexpensive and easily measured in the clinical settings. It is meaningful to consider more complications and risk of early recurrence in patients with high postoperative CRP through this study. If a patient’s CRP is high after surgery, it can be more carefully monitored and prepared for rapid action.

## Supplementary Information

**Additional file 1**.** Table S1**. Number of patients and C-reactive protein levels on each day after surgery.** Table S2**. Clinical risk factors and postoperative outcomes in patients with intestinal Behçet’s disease

## Data Availability

The raw data of the current study are not publicly available due to the protection of participants’ personal information but are available from the corresponding author on reasonable request.

## Abbreviations

ASA: aminosalicylic acids
AUC: area under the curve
BD: Behçet’s disease
CD: Crohn’s disease
CRP: C-reactive protein
DAIBD: disease activity index of BD
HR: hazard ratio
HLA: human leukocyte antigen
TNF: tumor necrosis factor
