# Supplementary material for: C-reactive protein is associated with postoperative outcomes in patients with intestinal Behçet’s disease
Source: BMC Gastroenterol. 2021 Oct 7;21:362. doi: 10.1186/s12876-021-01922-2 (PMC8496041; doi:10.1186/s12876-021-01922-2)
Supplement: Supplementary file 1 — Additional file 1. Table S1. Number of patients and C-reactive protein levels on each day after surgery. Table S2. Clinical risk factors and postoperative outcomes in patients with intestinal Behçet’s disease [file 12876_2021_1922_MOESM1_ESM.docx]

**Table S1. Number of patients and C-reactive protein levels on each day after surgery**

| POD | **Number of patients** | **CRP (mg/L)** |
| --- | --- | --- |
| 1 | 39 | 60.5 ± 77.1 |
| 2 | 18 | 85.7 ± 68.6 |
| 3 | 10 | 52.1 ± 54.6 |
| 4 | 7 | 71.0 ± 86.9 |
| 5 | 16 | 13.0 ± 41.4 |
| Total | 90 | 57.0 ± 71.2 |

*CRP* C-reactive protein, *POD* postoperative day

**Table S2. Clinical risk factors and postoperative outcomes in patients with intestinal Behçet’s disease**

|  | **Postoperative complication**  **(HR (95% CI),**  ***p*-value)** | **Clinical relapse**  **(HR (95% CI),**  ***p*-value)** | **Re-operation**  **(HR (95% CI),**  ***p*-value)** | **Re-admission**  **(HR (95% CI),**  ***p*-value)** |
| --- | --- | --- | --- | --- |
| Age | 1.04 (1.00–1.09)  *p* = 0.08 | 1.02 (0.99–1.05)  *p* = 0.14 | 1.00 (0.97–1.04)  *p* = 0.90 | 1.02 (0.99–1.05)  *p* = 0.26 |
| Sex | 0.70 (0.24–2.03)  *p* = 0.51 | 0.63 (0.30–1.34)  *p* = 0.23 | 0.89 (0.30–2.60)  *p* = 0.83 | 0.74 (0.33–1.66)  *p* = 0.47 |
| CRP | **1.01 (1.00–1.02)**  ***p* < 0.01** | **1.01 (1.01–1.02)**  ***p* < 0.01** | **1.01 (1.01–1.02)**  ***p* < 0.01** | **1.01 (1.01–1.02)**  ***p* < 0.01** |
| Albumin | 0.74 (0.27–2.02)  *p* = 0.56 | 0.77 (0.42–1.42)  *p* = 0.40 | 0.54 (0.23–1.28)  *p* = 0.16 | 0.72 (0.37–1.41)  *p* = 0.34 |
| Anti-TNF | 0.93 (0.28–3.06)  *p* = 0.91 | 0.53 (0.24–1.18)  *p* = 0.12 | 0.51 (0.16–1.59)  *p* = 0.25 | 0.63 (0.27–1.46)  *p* = 0.28 |
| Steroids | **4.36 (1.21-15.68)**  ***p* = 0.02** | 1.74 (0.85–3.54)  *p* = 0.13 | **3.31 (1.15–9.56)**  ***p* = 0.03** | 2.08 (0.97–4.48)  *p* = 0.06 |
| Typical Volcano ulcer | 0.13 (0.01–1.67)  *p* = 0.12 | **0.07 (0.01–0.45)**  ***p* = 0.01** | 0.19 (0.02–2.21)  *p* = 0.18 | **0.08 (0.01–0.49)**  ***p* = 0.01** |
| Intestinal fistula/abscess | 0.38 (0.07–2.11)  *p* = 0.39 | **0.13 (0.04–0.49)**  ***p* = 0.01** | 0.30 (0.06–1.41)  *p* = 0.29 | **0.16 (0.04–0.60)**  ***p* = 0.03** |
| DAIBD | 1.00 (0.97–1.02)  *p* = 0.73 | 1.01 (0.99–1.02)  *p* = 0.31 | 0.98 (0.96–1.01)  *p* = 0.13 | 1.00 (1.00–1.02)  *p* = 0.76 |

*CI* confidence interval, *CRP* C-reactive protein*, DAIBD* disease activity index of intestinal Behçet’s disease, *HR* hazard ratio, *TNF* tumor necrosis factor
